# The relationship between income level and road traffic deaths: an empirical analysis for 22 OECD countries

**DOI:** 10.1186/s12889-025-23726-9

**Published:** 2025-08-16

**Authors:** Yuksel Bayraktar, Serdar Aydin, Mehmet Firat Olgun, Ayfer Ozyilmaz, Metin Toprak

**Affiliations:** 1 Department of Economics, Ankara University, Çankaya, Ankara, 06590 Turkey; 2 Robinson College of Business, Georgia State University, Atlanta, GA 30303 USA; 3 Technology Transfer Office, Kastamonu University, Kuzeykent, Kastamonu, 37150 Turkey; 4 Department of Public Finance, Kırıkkale University, Kırıkkale, Yahşihan 71450 Turkey; 5 Department of Economics, Istanbul Sabahattin Zaim University, Kucukcekmece, Istanbul, 34060 Turkey

**Keywords:** Health economics, Road safety, Convergence, Income, Road accident

## Abstract

**Background:**

The increase in transportation and travel demands leads to the development of social welfare, and on the other hand, it may adversely affect socio-economic indicators such as death, injury, air pollution and budget deficit. Every day, thousands of people are killed, injured, or disabled due to road accidents around the world. The high cost of fatal and non-fatal road accidents to national economies is important in terms of policies to be implemented. This study aims to examine the relationship between road accidents and income levels in 22 OECD countries.

**Methods:**

Poisson Regression, Negative Binomial, and Quantile Regression Fixed Effect were used in models estimation. In addition, the convergence of traffic accident deaths for 34 OECD countries was investigated. Fractional frequency unit root test with structural break was used for convergence analysis.

**Results:**

The findings of the study show that there is an inverted U-shaped nonlinear relationship between road accident deaths and per capita income. In addition, while the increase in health expenditures reduces the number of deaths due to traffic accidents, the increase in alcohol consumption increases these deaths. The results obtained from the convergence analysis indicates that 21 OECD countries converge to the OECD average, but 13 countries do not converge.

**Conclusions:**

Infrastructure investments for road safety such as traffic lights, curves, wide highways should be effectively implemented by the public. Pedestrians or drivers who put road safety at risk should be given deterrent penalties when necessary. In addition, public awareness should be increased regarding traffic rules and life safety.

**Trial registration:**

Not applicable.

**Supplementary Information:**

The online version contains supplementary material available at 10.1186/s12889-025-23726-9.

## Background

Road accidents, which tend to increase day by day in the world due to the increasing road network, are one of the important socio-economic problems of countries. Many factors such as rapid urbanization, low safety standards, lack of supervision, the number of people driving tired, drugs, alcohol, speed and seat belts determine the occurrence of accidents [1]. Road accidents have a high share of the world’s deaths due to traffic accidents. About 1.3 million people die each year due to road traffic accidents. 93% of these deaths occur in low- and middle-income countries [2]. It is estimated that by 2030, road traffic injuries will cause approximately 3% of all deaths worldwide. This rate was 2% in 2002 [3].

The costs of road transport, which have a key role in maximizing social benefit, are divided into two groups as direct costs and indirect costs. Direct costs are the costs required to provide road transport services such as infrastructure, equipment and personnel. Road accidents and related deaths, travel delays due to road traffic jams, and air pollution are the leading indirect costs [4]. Among these costs, two main indicators should be particularly focused on. These are the deaths caused by road accidents and the economic dimension of these accidents. Because the economic cost of road accidents on national economies is significant. For example, road accidents cause costs corresponding to 1–3% of GNP in many countries [5]. In addition, it is estimated that fatal and non-fatal road injuries will cost the world economy approximately 1.8 trillion dollars in the 2015–2030 period [6].

In addition to the economic consequences of road accidents, one of the important questions is whether economic growth or income level has an impact on road safety. When viewed in the context of economic growth, in low-income countries, governments prioritize policies that focus on economic development. Policies designed to improve quality of life, including road safety, may become secondary. However, when a certain level of economic development is exceeded, policies for social welfare, including road safety, can find a basis for discussion and implementation. Therefore, as the level of economic development increases, governments invest more in ensuring road safety. In this context, in addition to the improvements in the road infrastructure, the increase in the use of safer transportation modes positively affects road safety [7–10].

The relationship between economic growth and road safety is discussed in some studies by separating them according to supply and demand dimensions. Accordingly, low-income societies invest less in road safety policies despite their need. Because the priority for these households is minimizing other public health risks and meeting their basic living needs. And high-risk modes of transport are more widely used than low-risk modes of transport to meet low-income travel demands. As income increases, individuals will tend to buy vehicles. This will bring traffic problems. But as income more, people are from begin prefer low-risk vehicles, possibly for comfort, prestige, and safety. There is a similar trend on the supply side. Low-income governments devote fewer resources to institutional arrangements for road safety. With economic growth, governments begin to turn to road safety measures to reduce traffic accidents and increase road safety. Ultimately, the improvement in the living conditions of the country allows reducing the number of traffic accidents [11, 12].

Statistical data on road safety according to the development levels of the countries support these highlighted views. For example, death rates from accidents are 3 times higher in low-income countries than in high-income countries. In this context, more than 90% of road traffic deaths and injuries occur in low- and middle-income countries. While there was no decrease in road traffic deaths in low-income countries between 2013 and 2016, there were decreases in the middle- and high-income countries [1, 5]. For example, in the last 60 years covering the period 1955–2015, road accident deaths have been falling regularly in high-income countries, but have increased or remained flat in low- and middle-income countries [13].

However, in some studies, the income-road safety relationship has been discussed from different perspectives and it is suggested that the direction of this relationship may be reversed by different mechanisms. For example, Noland and Laham [14] emphasized that Low-income individuals are more likely to walk more and drive less. This leads these individuals to assume less risk as drivers but more as pedestrians. And differently, Wegman et al. [15] Increasing income may lead households to own more than one vehicle. Vehicle ownership by young people, who are more likely to ignore traffic rules, may lead to more road accidents.

In addition to empirical studies and statistical reports emphasizing the linear relationship between road safety and income level, one of the prominent issues in this regard is the income level after which road safety will change. The focus of the studies on this subject is the Kuznets Curve. Accordingly, there is a Kuznets type inverted-U-shaped relationship between income level and road safety. In this context, road accidents and deaths increase in the early stages of economic development. Because the increase in the number of motor vehicles will pose an increasing threat to the pedestrian population. In addition, low-income communities allocate fewer resources to regulations to improve road safety. However, as the income level increases, the demand of society for policies that will reduce road safety increases, and road accidents tend to decrease with the effect of the relevant policies. For this reason, an inverted-U-shaped relationship is expected between road accidents and deaths due to these accidents and income levels [8–10, 16–21].

For example, Sirajudeen et al. [11] discussed the inverted U-shaped relationship between economic growth and road safety in the context of supply and demand. In terms of demand, individuals’ demands for road safety are quite limited at low levels of development, and the demand for more vehicle ownership will be strong. However, as the level of development increases, the demand for vehicles and technical products that will increase road safety will increase, which will lead to a decrease in the increasing number of road accidents and deaths in the first stage. A similar mechanism also applies to the supply channel. In the early stages, when development remains at a low level, governments may put road safety on the back burner and use their resources mostly in basic infrastructure investments. However, as the level of development increases, safety and social welfare issues also gain importance, and as a result of policies aimed at road safety, road accidents and deaths tend to decrease. As Makhubela [22] emphasizes, if the increased income is used for road infrastructure investments, traffic safety improves with improvements such as asphalt renewal, traffic lights, tunnels, viaducts and lighting, which reduces traffic accidents and deaths.

One of the transmission mechanisms in the effect of income on road safety is social awareness. Increasing income increases social awareness and education, causing individuals to be more conscious of the rules. This is one of the mechanisms that increase road safety in the long term [23]. In this contex, some studies suggest that there are positive relationship between education and road safety [14, 24]. Another transmission channel suggested is real markets. In this contex, Akdağ [25] emphasized that income can be a determinant on road safety by increasing traffic density through the trade volume channel [25].

When the literature is examined, to the best of our knowledge, there is no study examining the convergence of traffic accident-related deaths with Fourier unit root tests considering structural breaks for the OECD country group. Ignoring shocks originating from country dynamics may give misleading results in convergence analyses. Therefore, convergence analysis that takes structural breaks into account was preferred. This study provides important results for policy makers by presenting the convergence analysis with an effective and consistent estimator and also reveals the relationship between traffic accident-related deaths and GDP with different estimators for the OECD country group.

In this study, the relationship between road accidents and income in 22 OECD countries during the 1975–2018 period is examined within the scope of the Kuznets Curve. In this part of the study following the introduction, the empirical literature is included. In the second part, the data and method are presented, and in the third part, the empirical findings are discussed.

In some studies, growth rates are used in the relationship between economic growth and road safety, but this is completely outside the scope of our main discussion. Because the existence of countries with high growth rates despite being in the grouping of low-income countries makes it difficult to discuss the income level-road safety relationship on its own perspective. Studies using GDP per capita in the relationship between economic growth and road traffic accidents/deaths are at the focus of this study because they indirectly discuss the relationship between income and road safety.

Some studies focus economic growth-road safety and used GDP or per GDP for linear relationship. In these studies, some researcher claim that GDP increases road traffic death/injuries or accidents [26–28]. The general view in these studies is that with increasing income (relatively low increase) especially the young population may prefer motorcycles which are more dangerous, and demand for more travel. But some studies emphasize that GDP decreases road traffic death/injuries or accidents [20, 23, 29]. In these studies, the basic mechanisms that are emphasized are that individuals can purchase safer vehicles as their income increases, and that diffusion of knowledge and increased technical infrastructure and awareness are increased. From these stuedies, Jiang and Ma [29] investigated the relationship between traffic deaths and some macroeconomic indicators for the USA usin BO-CV-XG Boost model. There is a negative relationship between GDP and traffic deaths, and a positive relationship between poverty and traffic deaths. Therefore, income and road deaths are directly linked parameters. Because with income, individuals can perform vehicle maintenance, especially technical renewal related to security. Lack of resources will also bring security vulnerabilities. Akinyemi [20] analyzed the relationship between economic development and road safety in Nigeria. using ARDL approach. They found a linear and statistically significant relationship between economic development and road traffic accidents, deaths and injuries in Nigeria in the long run; accordingly, an increase in GDP reduces crashes and deaths in the long run but increases injuries.

In addiditon, Suphanchaimat et al. [26] discussed economic growth and road safety using negative binomial, spatial Durbin and fixed effects models and found a positive correlation between GDP and traffic injuries and deaths in Thailand. Region-specific conditions such as mountain roads, inadequate public enforcement, etc. may be decisive. In addition, after income reaches a certain level, individuals switch from more dangerous vehicles such as motorcycles to safer means of transportation such as automobiles, which increases road safety. When evaluated from another perspective, behaviors such as investing less in road safety and choosing risky transportation options (or neglecting technical maintenance) as a result of being focused on savings may play a role in this relationship. Bougueroua and Carnis [28] investigated the relationship between economic development and road safety for Algeria using the cointegration approach and vector error correction model. They found that the GDP per capita increases traffic accidents. Because increasing income also increases the demand for travel, and this increases traffic accidents. He et al. [23] examined the relationship between economic growth and road safety in Russia using multivariate fixed effects model. According to the study, increasing using gross regional product causes decrease in both road traffic deaths and accident death rates. To increase vehicle ownership and diffusion of knowledge may be decisive in obtaining these findings. Al-Reesi et al. [27] investigated the relationship between economic growth, motor vehicles and road traffic injuries in Oman. According to the study, GDP per capita is associated with the number of motor vehicles, which increases road traffic injuries and deaths. The fact that a significant portion of the population in Oman is under the age of 30, and that this age group is more vulnerable to road injuries, and that many accident victims in the country have difficult access to hospitals and depend more on the help of other individuals may be effective in these results. And Yannis et al. [30] found that as GDP increases in 27 European countries, deaths due to road accidents increase.

Some studies have examined the growth-road safety relationship using GDP by dividing countries/regions by income level or found the findings to be different depending on the region. For example. Li and Zhang [31] analyzed the relationship between regional economic development and road traffic safety in China with random effects model and Shapley value decomposition. GDP per capita negatively affects the number of traffic accidents and deaths in Eastern China, but this effect is insignificant in Western China for traffic-related injuries. In China overall, GDP per capita reduces traffic accidents but does not have a significant impact on accident-related deaths and injuries. Additionally, according to the Shapley value decomposition, GDP per capita has a important effect on road accidents and deaths. They claim that individuals in developed regions may also prefer public transportation more. This can reduce traffic injuries. Additionally, additional income may cause individuals to turn to safer vehicles. Peleckienė [32] analyzed the increase in motor vehicles and motor vehicle deaths by separating the EU countries according to their income levels using quantitative analysis. In the study, it was found that the rate of increase in motor vehicles in high-income EU countries is lower than the decrease in deaths per motor vehicle, while the rate of increase in motor vehicles in low-income countries is higher than the decrease in deaths per motor vehicle. Similarly, Bishai et al. [18] examined the relationship between income per capita and traffic accidents in 41 countries by grouping the countries according to their income levels. According to the study, as per capita income increases in low-income countries, the number of accidents, traffic accidents, and accident-related deaths increases. In high-income countries, an increase in per capita income reduces deaths from traffic accidents but does not reduce accidents or injuries.

Some studies discuss determinant of road safety or effect of different parameters on road safety and used GDP indirectly as control variables. For example, Zou et al. [33] investigated the impact of climate and weather conditions on fatal traffic accidents and also used GDP per capita. They found that there is no statistically significant relationship between GDP and fatal traffic accidents in California, but GDP increases fatal traffic accidents in Arizona. Makhubela [22] investigated the impact of some economic indicators on traffic accidents in Mpumalanga Province using regression analysis. According to the study, increasing GDP per capita reduces road accidents. Because increased income improves the living standards of individuals and provides them with additional financing opportunities for purchasing a new vehicle or vehicle maintenance. This is one of the mechanisms that can reduce traffic accidents. However, in the opposite case, especially in case of insufficient vehicle maintenance, it may lead to an increase in mechanical failures such as brake system failure and tire explosion. Rezaei et al. [34] investigated some determinants of road traffic deaths in Iran using time series analysis. According to study, GDP per capita negatively affects road traffic accident deaths in both the short and long run.

Researching road safety with socio-economic and demographic indicators, including income, Amiri et al. [35] investigated the impact of some socio-economic indicators on road safety in 365 cities in the State of California using Artificial Neural Networks and Principal Component Analysis. According to the study, mean household income shows an insignificant correlation with total fatals and injuries. Similarly, Ghiasvand et al. [36] examined the main socio-economic determinants of road traffic injuries among children in Iran with logistic regression analysis using 2010 statistical data. Household income was also used and income levels were divided into 4 groups. Children in the second and fourth income quartiles (higher income) are less likely to suffer road traffic injuries than children in the first quartiles Many parameters such as children’s access restrictions to motorcycles or bicycles, whether they reside in safe areas or not are decisive in the relationship between household income and children road safety. Noland and Laham [14] analyzed the relationship between socioeconomic and demographic characteristics and mortality rates using National Longitudinal Mortality Study Data the period of 1979–1989. According logistic regression results, there is no relationship between family income and motor vehicle accident deaths. The dual effect of vehicle ownership/lack of ownership may be effective in these results. For example, not being able to own a vehicle increases life safety due to not being a driver, but on the other hand, it can lead to a higher risk of accidents as a pedestrian. Gaygisiz [37] focused on some economic and cultural indicators that lead to road traffic accidents in 30 OECD members and 5 participating countries using Pearson product-moment correlations. Author found that there is a positive relationship between improvements in economic indicators (employment, decreasing income inequality and increasing per capita income) and traffic safety. Because in low-income countries, traffic safety is not a priority goal. However, as income increases, supportive policies such as road infrastructure, driver training and emergency medical services lead to increased road safety.

Many studies suggest a nonlinear relationship between income level and road safety. The prominent view in studies investigating the relationship between income and road accidents for different levels of income is that income increases road accidents up to a certain threshold and decreases them after a certain threshold. The basic mechanism here is that in the first stage, income increases vehicle ownership, and in the second stage, infrastructure investments, investments and training for road safety increase within the framework of maximizing social welfare [11, 22, 24, 38]. When we look at the studies that suggest an inverted-U- shaped, Sirajudeen et al. [39] investigated the relationship between the ratio of motorcycle deaths to passenger vehicle deaths and GDP per capita for 38 countries using panel linear regression analysis. They found that there is an inverted-U-shaped relationship between variables. Here, in a sense, the effect of the relative change between two means of transportation (motorcycle and passenger car) along with income on road safety is revealed. In this context, when GDP per capita is lower level, motorcycle deaths are higher than passenger car deaths, but as income increases, the impact of passenger cars becomes stronger. Because motorcycle owners are at a higher risk of fatal injuries than passenger car owners. Jadaan and Alqasem [40] investigated the relationship between GDP per capita and road deaths in Jordan. According to the study, road deaths increase due to the increase in motor vehicles in the early stages of economic growth; however, this relationship reverses after a certain turning point. The study emphasized that motorcycles are an effective factor on the increases in road deaths.

In addition, Sirajudeen et al. [11] found an inverse U-shaped relationship between GDP per capita and road death/road injury in 67 countries using fixed effects model. Because lower level of development, individuals aim to own more vehicles and thus road traffic increases; Road accidents may increase when both individuals and governments neglect road safety. However, after income reaches a certain level, individuals turn to safer vehicles and governments turn to policies aimed at road safety, thus road safety is positively affected by the high level of development. Wu et al. [38] investigated the impact of economic development and demographic factors on road safety (number of accidents, deaths and injuries) using gradient boosting decision tree model in Zhongshan, China. Accordingly, GDP per capita leads to an increase in traffic accidents until it reaches a critical value. The main reason for this is the effect of income on vehicle ownership. However, after income reaches a certain threshold, the effect of income turns negative. Because income reaching a certain level causes governments to focus on improving regulations for social life, including traffic safety, and this leads to a decrease in road accidents. Grimm and Treibich [41] investigated the socio-economic determinants of road traffic accidents for low- and middle-income countries using panel data analysis. They found an inverted U-shaped relationship between income and traffic accident deaths. Costs such as vehicle safety maintenance, helmets, driving training and safety equipment are difficult costs for low-income households. Therefore, income is a determinant of road safety. Anbarci et al. [24] investigated the relationship between income inequality and road accidents in 79 countries and also focused on the effect of income. They claim that there is a non-linear relationship between GDP per capita and traffic deaths. Accordingly, as GDP per capita increases, traffic fatality rates also increase, and after GDP exceeds the threshold level, income reduces road deaths. The reason for this may be that high-income individuals use more safer vehicles. Similarly, Pinheiro et al. [42], Clement et al. [12]; Law et al. [17]; Iwata [43], Moniruzzaman and Anderson [44], Kopits and Cropper [9], Van Beeck et al. [10]; Law [8], Law et al. [16] and Paulozzi et al. [19] found inverse U-shaped relationship, in Brazilian municipalities for traffic accidents; in Cameroon for road accidents; in 60 developed countries for road deaths; in China for traffic fatalities and injuries; in OECD countries for road accidents; in 88 countries for motor vehicle mortality; in OECD countries for traffic deaths; in both underdeveloped and developed 90 countries for road injuries,; in 25 countries for motorcycle accidents, respectively.

In the non-linear relationship, unlike these studies, Kaghazian et al. [45] focused on some economic factors affecting the shadow price of traffic accidents in the Iranian economy. According to ARDL analysis findings, as income increases, the shadow prices of road deaths increase, which means that income is in the same direction as road deaths in the first stage. Increasing income increases the number of vehicles, which causes traffic congestion and increases accidents and shadow costs of accidents. However, this relationship reverses as incomes and public infrastructure investments increase. In other words, there is a relationship between the variables similar to Kuznets’ inverted U curve.

In some studies, investigating the effects of different socio-economic indicators on road safety, GDP or GDP per capita was used as a control variable. In general view, GDP incrases road safety. Because more income causes more strong health system, more safety vehicle buying. In this contex, Navarro-Moreno et al. [46] investigated the effect of infrastructure investments on traffic accidents with injuries in 20 European countries using panel data analysis. They found that income negatively associated with traffic injuries. Zeng et al. [21] examined the relationship between socio-economic factors and traffic fatality rate in 31 provinces of China using Tobit model. They found a negative relationship between GDP per capita and traffic fatality rate. Calvo-Poyo et al. [47] also included GDP as a control variable in their study investigating the impact of economic resources on roads on road deaths for 23 European countries using panel data analysis. They claim that both road maintenance, protection expenditures, and GDP decrease mortality rates, but construction investments have the opposite effect. The factors that determine the effect of GDP on reducing road accidents may be the role of GDP in the health system, infrastructure and vehicle renewal. Li et al. [48] investigated the relationship between social media and road accidents in Hong Kong using ARDL and VECM model and also used GDP as a control variable. The findings suggest that there is a long-term relationship between GDP and road accidents. Nguyen-Hoang and Yeung [49] examined the impact of highway capital investments on highways and used per capita income as an indicator of economic conditions for 48 states in the USA. According to the study, road capital expenditures and road capital stock have a negative impact on traffic deaths. However, for income per capita, this effect is positive.

In addition this studies, Antoniou et al. [50] analyzed the relationship between GDP and unemployment and road traffic accident death risks with a macro panel time for 30 European countries using the Common Correlated Effects Mean Group estimator. Long-term elasticity was found to be positive and significant for 10 countries, and it is statistically insignificant for other countries. Additionally, when countries where the number of deaths is constant are excluded, the average elasticity is as high as approximately 1. Noland and Zhou [51] focused on the relationship between traffic deaths and economic recession at the state level in the USA. According to the study, which includes many parameters that may have an impact on deaths, as income increases, traffic deaths increase; As income inequality decreases, traffic deaths also decrease. And, increasing income also increases alcohol consumption, which will indirectly cause income to increase traffic deaths. When the findings are evaluated in general, the decrease in economic activities will also reduce the traffic death trend.

Wegman et al. [15] who indirectly discusses this relationship, analyzed how the 2008 global crisis affected traffic deaths in OECD countries. According to the study, economic recessions expressed by a decrease in GDP or a lower growth trend or unemployment reduce traffic deaths. Because the economic recession reduces the number of young drivers and may also lead to a decrease in speed and alcohol use.

Despite the large literature discussing income and road safety, the literature examining this relationship in the context of convergence is limited. Different results were found in convergence analyses and different transmission channels were emphasized in this context. For example, according to Nghiem et al. [52] and Castillo-Manzano et al. [53] as countries with higher rates of road traffic crashes see and turn to technologies to reduce traffic crashes in other countries, convergence on road safety may occur over time between these countries. Castillo-Manzano et al. [53] discussed the convergence of road accident fatalities in EU countries. They found that convergence in deaths due to road accidents across the panel. Accordingly, in countries with high road accident deaths, death rates decrease over time, and the disparity between road accident deaths among countries decreases over time. Similarity and harmonization efforts in basic policies for road safety in EU countries play an important role in these findings. Nghiem et al. [52] investigated the convergence of road accident deaths to 23 OECD countries. The findings show that road accident deaths do not converge, but there is convergence among country subgroups. Additionally, some determinants of road traffic deaths were investigated with a fixed effects model and an inverted U-shaped relationship was found between GDP per capita and traffic death rates.

## Methods

This study aims to investigate the relationship between road accidents and income levels. In addition, this relationship was also examined in the context of convergence analysis. For regression analyses, data from 22 OECD countries were used, and for convergence, data from 34 OECD countries were used. In the convergence analysis, the convergence of road accident deaths in OECD countries to the OECD average was investigated using stochastic convergence method. The study covers the period 1975–2018 but for convergence analyses this period is 1994–2020. For model estimation, Poisson Regression, Negative Binomial, and Quantile Regression Fixed Effects estimators were used. When the assumption of normality is not satisfied, since the OLS estimator causes biased estimations, Poisson Regression Fixed Effects, and Negative Binomial Fixed Effects estimators were used in the model estimation in which the dependent variable is count data.

In the other model, in which the dependent variable was not count-data, the Quantile Regression Fixed Effects estimator, which is an estimator sensitive to this situation, was used when the dependent variable has extreme values. Information on the variables used in the model is given in Table 1.

**Table 1:** Description of variables and data source

Variables	Definition	Source
Fatality	Road accidents (Deaths, number)	OECD
lnFatality	Road accidents (Deaths, number)	OECD
lnGDP	GDP per capita (constant 2010 US$)	World Bank
lnHS	Health spending (Total, % of GDP)	OECD
lnAC	Alcohol consumptionTotal, Litres/capita (aged 15 and over)	OECD

ln indicates that the variables are included in the model by taking the natural logarithm

The stochastic convergence method was used in the convergence analysis. In this context, the transformation for the data set is as in Eq. (1):

1 $$\:{y}_{t}^{i}=ln\left(\raisebox{1ex}{${Death}_{t}^{i}$}\!\left/\:\!\raisebox{-1ex}{${AverageDeath}_{t}$}\right.\right)$$

For the stationarity of the new series obtained, Bozoklu et al. [54] structural break fractional frequency unit root test was used. The stationarity of the series indicates the presence of convergence.

Descriptive statistics of the variables are shown in Table 2.

**Table 2:** Descriptive statistics

Variables	Obs.	Mean	Std. Dev.	Min	Max	Jarque-Bera
Fatality	968	4392.85	8789.16	4	5109	9520.65*** (0.000)
lnFatality	968	7.220	1.622	1.386	10.841	35.972*** (0.000)
lnGDP	968	10.366	0.567	7.876	11.430	549.249*** (0.000)
lnHS	968	1.988	0.338	0.398	2.836	616.045*** (0.000)
lnAC	968	2.130	0.540	0.182	2.975	2062.30*** (0.000)

***Indicates that the series is not normally distributed at the 1% significance level

The number of deaths due to road accidents was used as the dependent variable in the study. Since the dependent variable is count data, model estimation was made by the negative binomial regression method from count-data regression models. Because in case of over dispersion, the Poisson regression method causes biased and inconsistent estimates. Therefore, the results of the Poisson regression model are tabulated for comparison.

Poisson regression is used when a dependent variable is a count number. This method is based on the Poisson distribution and the mean-variance being equal to each other. The Poisson distribution is a single parameter distribution. This parameter is λ and is equal to the mean and variance. λ has to be positive. It is possible to express this parameter exponentially [55].

2 $$\:\lambda\:=\text{e}\text{x}\text{p}({\beta\:}_{1}+{\beta\:}_{2}{x}_{2}+\dots\:..+{\beta\:}_{k}{x}_{k})$$

The assumption that the mean and variance are equal in the Poisson regression model is often not met. The variance turns out to be larger than the mean [56]. In this case, the negative binomial fixed effects model is more consistent. This model also takes into account heterogeneity. The assumption that variance and mean are equal is not valid in this model [55, 57]. The negative binomial fixed effects estimator was developed by Hausman et al. [56]. The negative binomial fixed effects model can be defined as follow:

3 $$\:{\lambda\:}_{it}=\text{exp}\left({a}_{i}+{x}_{it}^{{\prime\:}}\beta\:\right)$$

4 $$\:\text{ln}\left({\lambda\:}_{it}\right)={a}_{i}+\beta\:{X}_{it}{\prime\:}\:\:\:\:i=1,\dots\:.,N\:\:\:\:t=1,\dots\:..,T$$

Hausman et al. [56] defined the equation $$\:{\theta\:}_{i}=\raisebox{1ex}{${a}_{i}$}\!\left/\:\!\raisebox{-1ex}{${\varphi\:}_{i}$}\right.$$ taking into account $$\:{a}_{i}$$ and the negative binomial overdispersion parameter $$\:{\varphi\:}_{i}$$ which shows specific effects. The most important limitation of the model is that $$\:{a}_{i}$$ and $$\:{\varphi\:}_{i}$$ are defined up to the ratio $$\:\raisebox{1ex}{${a}_{i}$}\!\left/\:\!\raisebox{-1ex}{${\varphi\:}_{i}$}\right.$$ [57]. Considering Eq. (3), the mean and variance for the negative binomial fixed effects estimator are as follows.

5 $$\:E\left({Y}_{it}\right)=\frac{{a}_{i}{\lambda\:}_{it}}{{\varphi\:}_{i}}$$

6 $$\:Var\left({Y}_{it}\right)=\frac{{a}_{i}{\lambda\:}_{it}}{{\varphi\:}_{i}}\:\left(\frac{1+{a}_{i}}{{\varphi\:}_{i}}\right)$$

The conditional maximum likelihood function is as follows [57, 58]:

7 $$\begin{aligned} \:\text{Pr}\left[\left.{y}_{i1},\dots\:\dots\:,{y}_{iT\:}\right|\sum\:_{t=1}^{T}{y}_{it}\right]=&\left(\prod\:_{t}\frac{{\Gamma\:}\left({\lambda\:}_{it}+{y}_{it}\right)}{{\Gamma\:}\left({\lambda\:}_{it}\right){\Gamma\:}\left({y}_{it}+1\right)}\right)\\&x\frac{{\Gamma\:}\left(\sum\:_{t}{\lambda\:}_{it}\right){\Gamma\:}\left(\sum\:_{t}{y}_{it}+1\right)}{{\Gamma\:}\left(\sum\:_{t}{\lambda\:}_{it}+\sum\:_{t}{y}_{it}\right)} \end{aligned}$$

As seen in Table 3, the OLS estimator gives biased results when the dependent variable does not have a normal distribution (extreme values). In the case of extreme values, it is more appropriate to use the quantile regression method, which is less sensitive to extreme values [59]. However, one of the problems encountered in the application of the quantile regression method is ignoring the unobservable individual heterogeneity. However, quantile regression with fixed effects takes this issue into account [60]. Considering that the deaths due to accidents may differ between the countries studied, the analysis was made with the quantile regression fixed effects method. The moments of method quantile regression with fixed effect method developed by Machado and Santos Silva [61] allows individual effects to affect the entire distribution.

In the panel data model with units and time dimensions, the position-scale estimation model of conditional quantiles $\:{Q}_{Y}\left(\tau\:|X\right)$ is defined as follows.

8 $$\:{Y}_{it}={a}_{i}+{X}_{it}^{{\prime\:}}\beta\:+\left({\delta\:}_{i}+{Z}_{it}{\prime\:}\gamma\:\right){U}_{it}$$

Where Pr$\:\left\{{\delta\:}_{i}+{Z}_{it}^{{\prime\:}}\gamma\:>0\right\}=1$. $\:{\left(\alpha\:,\:{\beta\:}^{{\prime\:}},\:\delta\:,\:{\gamma\:}^{{\prime\:}}\right)}^{{\prime\:}}\:$are unknown parameters. ($\:{a}_{i},\:{\delta\:}_{i}$), i=1,….,n parameters show individual i fixed effects. $\:{X}_{it}$is the vector of independent variables. $\:{U}_{it}$ represents unobservable random variables. $\:{U}_{it}$ and $\:{X}_{it}\:$are unrelated. Z is a defined vector k of differentiable transformations of the components of X with I element [61].

9 $$\:{Z}_{I}={z}_{I}\left(X\right),\:\:\:I=1,\dots\:..,k$$

Model (10) implies that;

10 $$\:{Q}_{Y}\left(\tau\:|{X}_{it}\right)=\left({a}_{i}+{\delta\:}_{i}q\left(\tau\:\right)\right)+{X}_{it}^{{\prime\:}}\beta\:+{Z}_{it}{\prime\:}\gamma\:q\left(\tau\:\right)$$

Where $\:{a}_{i}\left(\tau\:\right)={a}_{i}+{\delta\:}_{i}q\left(\tau\:\right)$ is the distributional effect at τ [61].

Fractional frequency unit root test with structural break developed by Bozoklu et al. [54] was used in convergence analysis. This method allows frequency values to take values between 0 and 5, including fractional ones. In the first stage of the test, the following model is estimated [54].

11 $$\begin{aligned}\:\varDelta\:{y}_{t}=&\rho\:{y}_{t-1}+{\beta\:}_{1}+{\beta\:}_{2}trend\\&+{\beta\:}_{3}sin\left(2\pi\:kt/T\right)\\&+{\beta\:}_{4}cos\left(2\pi\:kt/T\right)+{u}_{t} \end{aligned}$$

Here t is the trend, T is the number of observations. In order to find the optimum k value, values are given starting from 0.1 with 0.1 increments up to 5, and the k value that gives the minimum SSR is determined as the optimum lag value. This test is used if trigonometric terms are meaningful. In testing the significance of trigonometric terms [54];

12 $$\:{\beta\:}_{3}={\beta\:}_{4}=0$$

The hypothesis is being tested. The basic hypothesis is trigonometric terms are insignificant/Fourier function is insignificant. F test is used to test trigonometric terms. The F test statistic is compared with the table values of Enders and Lee [62]. If the F test statistic is greater than the table value, it is decided that the trginometric terms are significant. If the trigonometric terms are insignificant, traditional ADF unit root test results should be consulted. If the trigonometric tests are significant, the stationarity of the series is tested with the FADF test statistic. The FADF test statistics are decided by comparing the table values in [54].

## Results

The results of cross-section dependence, unit root, homogeneity and cointegration tests are given in Table 3.

**Table 3:** Cross-sectional dependency, unit root and cointegration test results

Cross-Sectional Dependency (for variables)				
Variables	BP (T > *N*)		LmAdj	
lnFatality	426.634*** (0.000)		355.590*** (0.000)	
lnGDP	1515.823*** (0.000)		424.204*** (0.000)	
lnHS	603.079*** (0.000)		424.204*** (0.000)	
lnAC	261.241*** (0.000)		383.569*** (0.000)	
CIPS Unit Root Test				
Constant	Constant + Trend		First Difference	
lnFatality	−2.217**	−2.7559**		−4.8067***
lnGDP	−2.1073	−2.5261		−3.9539***
lnHS	−2.2864**	−2.4669		−4.6818***
lnAC	−1.9010	−2.5988		−4.0517***
LM_Adj Cross-Sectional Dependency Test Results (for residuals)				
LM	9.494 (0.000)			
Homogeneity Test Results				
Swamy S.	1035.32*** (0.000)			
$\tilde\triangle$	1791.78*** (0.000)			
${\tilde\triangle}_{adj}$	1949.85*** (0.000)			
$\hat\triangle$	71.40*** (0.000)			
${\hat\triangle}_{adj}$	1.779** (0.037)			
Westerlund Cointegration Test				
DH_g	0.944 (0.173)			
DH_p	1.701** (0.044)			

Values in parentheses indicate the probability value

Breusch and Pagan [63] Test and LM_Adj test developed by Pesaran, Ullah and Yamagata [64] were used to test cross-section dependence. Cross-section test results show that there is cross-section dependence in all series. Therefore, the CIPS Unit Root test developed by Pesaran [65], which takes into account the cross-section dependence, was used to test the stationarity of the series. According to the CIPS unit root test results, while accident-related deaths and health expenditures are stationary in level value, per capita income and alcohol consumption are stationary at the first difference. When the homogeneity test results are examined, it is seen that the slope parameters are heterogeneous. Westerlund [66] cointegration test was used to examine the long-term relationship between the series since the series are stationary at different degrees and take into account heterogeneity. The results obtained from the Westerlund cointegration analysis show that there is a cointegration relationship for the panel (DH_p).

The estimation results are given in Table 4.

**Table 4:** Analysis results

Variable	Poisson Regression Fixed Effect	Negative Binomial Fixed Effect	Quantile Regression Fixed Effect				
			20th Quantile	40th Quantile	60th Quantile	80th Quantile	
lnGDP	5.4231*** (0.000)	8.7327*** (0.000)	9.6673*** (0.000)	9.6273*** (0.000)	9.5968*** (0.000)	9.5709*** (0.000)	
lnGDP2	−0.2843*** (0.000)	−0.4523*** (0.000)	−0.5036*** (0.000)	−0.5014*** (0.000)	−0.4997*** (0.000)	−0.4982*** (0.000)	
lnHS	−0.3322*** (0.000)	−0.8389*** (0.000)	−0.8334*** (0.000)	−0.7750*** (0.000)	−0.7304*** (0.000)	−0.6925*** (0.000)	
lnAC	0.2886*** (0.000)	0.2204*** (0.000)	0.4712*** (0.000)	0.4926*** (0.000)	0.5089*** (0.000)	0.5228*** (0.000)	

***<0.01. The value in parentheses shows the probability values. The natural logarithm of the number of deaths due to road accidents were taken in Quantile regression method

According to Table 4, income per capita (lnGDP) is positive in all estimation methods; the square of per capita income (lnGDP^2^) is negative and statistically significant. The estimation results show that there is an inverted-U-shaped Kuznets relationship. Therefore, an increase in per capita income up to a certain level increases deaths due to road accidents, but after a certain income level, deaths due to road accidents decrease. The per capita income level, where deaths due to road accidents started to decrease, was calculated as $15.578 using the negative binomial fixed effects method for the country group under consideration. According to the quantile regression fixed effect estimation results, this turning point was calculated as $14.738 in countries with low accidental deaths (20th quantile), but $14.845 in countries with high accidental deaths (80th quantile). The quantile regression results show that the turning point is lower in countries with low accident-related deaths than in countries with high accident-related deaths. Law et al. [17] estimated the turning point in developed countries as $13.767. According to the Poisson regression fixed effects estimation result presented as a comparison, the per capita income level at which the turning point occurs is $13.872.

Considering the other control variables included in the model, negative binomial fixed effects and quantile regression fixed effect estimation results show that all variables are statistically significant. The effect of the share of health expenditures in GDP on the number of deaths due to road accidents is negative and has a statistically significant effect at the 1% level. Therefore, as health expenditures increase, the number of deaths due to road accidents decreases. It is similar to the results of Ali et al. [67] that health expenditures have a negative effect on road accidents. Looking at the quantile regression fixed effects estimation results, a 1% increase in health expenditures reduces accidental deaths by 0.833% in countries where accident-related deaths are low. Alcohol consumption (lnAC) has a positive and statistically significant effect on road accident deaths at the 1% significance level. Quantile regression estimation results show that a 1% increase in alcohol consumption increases accidental deaths by 0.52% in countries with high accident-related deaths. Alcohol consumption has been seen as a determining variable on road accident deaths in many studies. Moskowitz [68] showed that a certain level of alcohol in the blood has a negative effect on the abilities of drivers. The studies of Law et al. [17] and Bishai et al. [18] also showed that alcohol consumption has a positive effect on road accident deaths.

The results of the convergence analysis for 34 OECD countries are given in Table 5.

**Table 5:** Convergence analysis results

Country	Frq.	Min. SSR	F Test Stats.	Op. lag	FADF Test Stats.	ADF Test Stats.
Australia	0.8	0.038	22.077^a^	1	−6.177***	0.049
Austria	0.1	0.077	2.658	1	−3.226	−3.450**
Belgium	2.3	0.067	4.292	1	−2.438	−2.170
Canada	0.8	0.027	7.551^c^	1	−3.535	−1.220
Chile	0.8	0.062	17.764^a^	3	−4.668***	−0.816
Czech Republic	1.2	0.085	6.951^c^	1	−5.683***	−3.777***
Denmark	4.2	0.248	2.879	5	−2.418	−2.527
Estonia	0.3	0.463	2.243	6	−1.894	−2.702*
Finland	0.6	0.048	12.018^a^	1	−4.913***	−0.956
France	1.7	0.086	5.881	6	−1.301	−1.490
Germany	1.5	0.037	7.549^c^	6	−2.415	−2.469
Greece	1.8	0.071	2.822	6	0.614	−1.315
Hungary	2.2	0.062	1.330	6	−1.332	−1.996
Iceland	2	2.925	3.700	1	−3.391	−5.203***
Ireland	0.4	0.125	9.570^b^	6	−3.877*	−0.921
Israel	0.1	0.131	3.593	1	−3.825	−3.331**
Italy	3.4	0.051	6.381^c^	1	−3.027**	−2.502
Japan	1.8	0.028	13.192^a^	5	−4.264***	−2.519
Lithuania	1.1	0.200	15.645^a^	5	−4.658***	−1.407
Luxembourg	1.8	0.325	7.019^c^	2	−5.398***	−3.683**
Latvia	1	0.149	8.934^b^	2	−4.446***	−2.034
Netherlands	0.5	0.057	15.553^a^	4	−5.195***	−0.552
New Zealand	0.5	0.139	8.917^b^	3	−4.004**	−0.621
Norway	0.1	0.274	5.457	1	−3.239	−2.150
Poland	1.1	0.069	5.911	3	−3.608	−2.336
Portugal	0.1	0.190	3.608	1	−3.112	−1.630
South Korea	1.7	0.096	38.002^a^	3	−5.673***	−1.873
Spain	1	0.029	40.602^a^	6	−4.413**	−0.584
Slovakia	1.2	0.225	6.389^c^	5	−3.740**	−3.344**
Slovenia	0.1	0.175	3.614	1	−2.882	−2.037
Sweden	0.1	0.149	1.430	0	−3.999	−4.046***
Switzerland	2.3	0.163	4.222	1	−2.892	−2.284
United Kingdom	1.8	0.026	13.395^a^	1	−4.454***	−2.638*
United States	0.1	0.021	4.127	6	−2.092	1.380

^a,b,c shows that the trigonometric terms are statistically significant at 99%, 95%, 90%confidence levels^

^***,**,*Indicate that the series are stationary at 1%, 5%-10%significance level. For the ADF unit root test, the maximum lag length was 2 and the Schwarz information criterion was used to determine the optimum lag length^

According to the convergence analysis findings, trigonometric terms are statistically significant in 18 countries. For this reason, fractional frequency unit root test with structural break was used to determine the stationarity of these series. However, for countries where trigonometric terms are not significant, the traditional ADF unit root test was used. According to the results of the fractional frequency unit root test with structural break, deaths due to road accidents in Australia, Chile, Czech Republic, Finland, Ireland, Italy, Japan, Lithuania, Luxembourg, Latvia, Netherlands, New Zealand, South Korea, Spain, Slovakia, United Kingdom converges to the OECD average. In addition, according to the results of the ADF unit root test, in which trigonometric terms are not significant, it is seen that Austria, Estonia, Iceland, Israel and Sweden converge to the OECD average In total, 21 OECD countries converge to the OECD average. According to the general findings, it can be said that the policies aimed at preventing deaths due to road accidents in OECD countries are effective.

## Discussion

This study investigates the relationship between road safety and income for 22 OECD countries. Fixed effect quantile regression, poisson regression and negative binomial models are used for coefficient estimation and the study covers the period 1975–2018. In addition, the convergence of traffic accident deaths for 34 OECD countries investigated using fractional frequency unit root test with structural break. Due to the accessibility of the data, different country groups were examined for coefficient estimation and convergence analyses. According to the study, there is an inverted-U-shaped relationship between deaths due to road accidents and GDP per capita.

In studies investigating the relationship between GDP and road safety indicators, it is generally observed that there is an inverted-U shaped relationship between the variables. For example, in recent studies, Sirajudeen et al. [39], Jadaan and Alqasem [40]; Sirajudeen et al. [11]; Wu et al. [38] etc. found similar findings for different countries. For OECD countries, Moniruzzaman and Anderson [44] and Van Beeck et al. [10] reached similar results. Studies have explained this inverted-U shaped relationship between economic growth or more specifically GDP and road safety through different mechanisms. For example, according to Sirajudeen et al. [39], low income growth leads individuals to buy motorcycles, which have a higher risk of accidents, instead of safe vehicles. This negatively affects road safety by allowing individuals to buy less safe means of transportation. However, when income exceeds a certain threshold, individuals turn to safer cars. Similarly, Jadaan and Alqasem [40] also linked this relationship to motorcycles. However, Wu et al. [38] emphasized the role of the public in this relationship. The authors claim that the security-reducing effect of income in the first stage is that income increases vehicle ownership. governments only turn to policies for social life, including traffic safety, after income reaches a certain level, so at high income levels, road accidents/deaths and injuries tend to decrease. According to Grimm and Treibich [41], costs such as vehicle safety maintenance, helmets, driving training and safety equipment are difficult costs for low-income households, therefore, income is a determinant of road safety. Anbarci et al. [24] also attributed this to high-income individuals purchasing safer vehicles.

In summary, individuals with low income levels cannot renew technical improvements such as automatic alarm systems, airbags and rear view cameras, which are decisive in driving safety, due to lack of resources. Therefore, insufficient income directly affects fatal injuries [29]. In contrast, low-income individuals drive older vehicles, speed more, and are more careless with their seat belts [14].

In addition to these studies, He et al. [23] emphasized that one of the transmission mechanisms of the effect of income on road safety is social awareness. Because increasing income increases social awareness and education, making individuals more conscious of the rules. This is one of the mechanisms that increases road safety in the long term.

In the second stage of the study, the convergence between deaths due to road accidents was analyzed for 34 OECD countries. For convergence analysis, fractional frequency unit root test with structural break was used. Analysis results revealed that 21 OECD countries converged to the OECD average, but there was no convergence in 13 countries. The question of why convergence occurs is answered by Nghiem et al. [52] and Castillo-Manzano et al. [53] explained it as technology transfer. Countries with higher road traffic accidents adopt road safety technologies from countries with lower accident numbers, thus converging road safety levels. Another emphasis is the convergence of policies, for example, within the context of harmonization efforts for EU countries.

## Conclusion

In this study, the relationship between road safety and income level was investigated in 22 OECD countries for the 1975–2018 period. Findings indicate that income level is statistically significant and positive on road accident deaths, while the square of income is negative and statistically significant. Therefore, inverted-U-shaped Kuznets type relationship was found to be valid between road accidents and income level. In other words, as the income level increases, the deaths due to road accidents increase at the beginning, but after the income level reaches a certain level, the deaths due to road accidents decrease. After which level the income level decreases the deaths due to road accidents differed according to the methods used. The per capita income level, where deaths due to road accidents started to decrease, was calculated as $15,578 using the negative binomial fixed effects method for the country group under consideration. According to the quantile regression fixed effect estimation results, this turning point was calculated as $14,738 in countries with low accidental deaths (20th quantile), but $14,845 in countries with high accidental deaths (80th quantile).

The study also investigated the convergence of road accident deaths to the OECD average in 34 OECD countries during the 1994–2020 period. According to the results of the convergence analysis, road accident deaths converge to the OECD average in 62% of OECD countries.

The high cost of fatal and nonfatal road use to national economies reveals the importance of road safety policies. In this context, policymakers should focus on the policies aiming to increase road safety, i.e. healthy urbanization policies, adequate inspection, high safety standards, increasing the quality of regulatory institutions, measures to prevent drug and alcohol consumption, and measures based on creating awareness for driving safety (not speeding, using seat belts, public service announcements), improvement of road infrastructure, safer transportation modes, the speed of access to accidents and the quality of medical care, and the regulation of traffic mobility.

In addition, the use of public transport should be encouraged in densely populated areas, thus minimizing traffic congestion. Considering the share of motorcycles in road accidents, regulatory policies and penalties for these vehicles, especially helmets, should be implemented.

## Supplementary Information

Supplementary Material 1.

## Data Availability

No datasets were generated or analysed during the current study.
