# Supplementary material for: The relationship between income level and road traffic deaths: an empirical analysis for 22 OECD countries
Source: BMC Public Health. 2025 Aug 16;25:2809. doi: 10.1186/s12889-025-23726-9 (PMC12357409; doi:10.1186/s12889-025-23726-9)
Supplement: Supplementary file 1 — Supplementary Material 1. [file 12889_2025_23726_MOESM1_ESM.docx]

# Appendix

Appendix 1: 22 OECD Countries

| Australia | South Korea |
| --- | --- |
| Austria | Netherlands |
| Belgium | New Zealand |
| Canada | Norway |
| Denmark | Portugal |
| Finland | Spain |
| Germany | Sweden |
| Iceland | Switzerland |
| Ireland | Turkey |
| Israel | United Kingdom |
| Japan | United States |
